# Comparison of Treatment Efficacy and Survival Outcomes Between Asian and Western Patients With Unresectable Gastric or Gastro-Esophageal Adenocarcinoma: A Systematic Review and Meta-Analysis

**DOI:** 10.3389/fonc.2022.831207

**Published:** 2022-03-07

**Authors:** Zhening Zhang, Zining Liu, Zeyang Chen

**Affiliations:** ^1^ Department of General Surgery, Peking University First Hospital, Beijing, China; ^2^ Department of Gastrointestinal Oncology, Key Laboratory of Carcinogenesis and Translational Research, Peking University Cancer Hospital and Institute, Beijing, China; ^3^ Department of Gastrointestinal Cancer Center, Key Laboratory of Carcinogenesis and Translational Research, Peking University Cancer Hospital and Institute, Beijing, China

**Keywords:** gastric cancer, gastro-esophageal adenocarcinoma, immunotherapy, anti-angiogenic therapy, anti-HER2 therapy, overall survival, progression-free survival, regional difference

## Abstract

**Background:**

Gastric cancer and gastro-esophageal adenocarcinoma are geographically heterogeneous diseases. Previous studies suggested that Asian and Western patients with late-stage gastric or gastro-esophageal adenocarcinoma possess distinct survival outcomes. However, the interregional differences of multiple systemic therapies in unresectable diseases have not been comprehensively described.

**Materials and Methods:**

We searched PubMed-MEDLINE, Embase, Web of Science and Cochrane Library from inception to 31 October 2021 and reviewed major conference abstracts for controlled trials of systemic therapies in unresectable gastric or gastro-esophageal adenocarcinoma that reported hazard ratios stratified by geographical region. The primary measurements were overall survival and progression-free survival. The pooled hazard ratios and 95% confidence intervals for overall survival and progression-free survival in Asian and Western populations were calculated using a random effect model. A linear regression model was adopted to compare the overall survival and progression-free survival between Asian and Western patients.

**Results:**

A total of 9033 patients from 20 studies were included for analysis. Immunotherapy was associated with an improvement in the overall survival for both Asian (hazard ratio, 0.80; 95% confidence interval, 0.65–0.98) and Western (hazard ratio, 0.90; 95% confidence interval, 0.81–1.00) patients, with no significant difference between the two groups (P = 0.32). Trends of survival benefit with anti-HER2 therapy and anti-angiogenic therapy versus control were observed in both Asian and Western patients, although statistical significance was not denoted. Subgroup analyses yielded a statistically superior overall survival of Asian versus Western patients in trials that investigated first-line immunotherapy (P = 0.04). Due to the linear regression analyses with scatter plot graphs, Asian patients showed a higher overall survival, but not progression-free survival, than Western patients irrespective of treatment type.

**Conclusion:**

Asian and Western patients with unresectable gastric or gastro-esophageal adenocarcinoma show similar responses to systemic therapies with limited interregional differences. Exceptionally, first-line immunotherapy could elicit superior survival among Asian populations. In addition, Asian patients with gastric or gastro-esophageal adenocarcinoma display a superior OS compared with Western counterparts.

## 1 Introduction

Gastric cancer (GC) and gastro-esophageal adenocarcinoma (GEA) are the fourth cause of cancer mortality worldwide (1). The incidence of GC/GEA varies across regions, with the highest estimated rate in Asia/Pacific and the lowest in North America (2). Despite a decline in global incidence within the past few decades, a substantial proportion of unresectable GCs remain incurable and portend dismal prognosis. For a long time, the mainstay chemotherapy regimen for unresectable GCs encompasses first-line platinum-based doublet and second-line taxanes (3–5). Nevertheless, treatment modalities for advanced/metastatic GC have undergone drastic evolution in recent years. Novel medications emerge exponentially, including immunotherapy, which predominantly exerts immune checkpoint blockade, antiangiogenic therapy, which ameliorates vascular remodeling, and growth factor receptor-targeted therapy, which counteracts aberrant cancer signaling, equipping oncologists with a vast number of robust weapons against late-stage GCs (6–8).

Notably, GC/GEA are highly heterogeneous diseases regarding geographic locality. According to previous studies, Asian (comprised of Japan, China, and South Korea) and Western (mainly Caucasians from North America or West/North Europe) GC patients have distinct prognoses even if balanced by stage. Asian patients are reported to possess longer PFS and OS according to subgroup analyses of multinational RCTs. By contrast, Western patients suffer from shorter survival and prone to show poorer responses to systemic therapies (9–14). It has been considered that the variation of both genetic and sociocultural factors contributes to the disparities. In terms of molecular patterns, somatic gene mutation or amplification rates in oncogenes such as HER2, EGFR, and KRAS are similar across regions. Nevertheless, Western GCs present molecular signatures regarding inflammation and T cell function, while Asian GCs do not (15–17). Taking into account the selective nature of targeted therapies and immunotherapy, we reasonably infer that the efficacy of various systemic therapies might differ between Asian and Western populations.

Uncovering the discrepancies of survival outcomes and treatment efficacy is critical for clinicians, as they can identify beneficiaries more efficiently and might develop strategies to eliminate disparities. However, the variety and volume of existing studies restrict clinicians to precisely make a judgment. Therefore, in this research, we aimed to quantificationally evaluate whether various systematic therapies exhibit different efficacies in Asian and Western patients with unresectable GC or GEA, measured in terms of OS and PFS. We also attempt to verify the correlation between survival parameters and geographic locality.

## 2 Materials and Methods

### 2.1 Literature Review and Inclusion Criteria

An electronic literature search with language limited to English was conducted utilizing PubMed-MEDLINE, EMBASE, Web of Science and the Cochrane Library to identify clinical trials published from inception to October ^31^, 2021. In addition, we reviewed conference abstracts from the Annual Meetings of the American Society of Clinical Oncology (ASCO) and the European Society of Medical Oncology (ESMO) during the last 20 years (2001-2021). Potentially relevant studies were retrieved with their references manually checked. Separately published subgroup analyses were also screened as the supporting data source. Detailed search algorithms are presented in **Supplementary Table 1**. Our meta-analysis followed the Preferred Reporting Items for Systematic Reviews and Meta-analyses (PRISMA) guidelines (18).

Eligibility criteria were as follows: 1) randomized or nonrandomized controlled clinical trials that recruited both Asian and Western patients (defined as patients from North America, Oceania or West/North Europe) with pathologically confirmed unresectable GC or GEA; 2) investigated the clinical benefit of systematic therapies (including chemotherapy, targeted therapy, immunotherapy or any of the combinations); and 3) reported subgroup survival outcomes (OS or PFS) stratified by geographical regions (including Asia). Exclusion criteria were as follows: 1) trials with single-arm design; 2) either Asian or Western participants were not enrolled; 3) subgroup analyses were lacking; 4) non-systemic interventions were investigated (e.g., local radiotherapy, debulking surgery). For trials that did not report subgroup outcomes, we tried to contact the corresponding author for integrated data.

### 2.2 Overall Design of the Meta-Analysis

The analyses contained two parts. In the first part, a meta-analysis investigating the interregional differences in treatment efficacy was performed. In the second part, OS and PFS between Asian and Western populations with unresectable GC/GEA were compared.

### 2.3 Data Extraction

Data extraction was performed independently by two investigators, ZZ and ZL. Discrepancies were consulted and resolved by the senior author ZC. Trial name, name of first author, year of publication, treatment regimen, treatment line and the number of participants in each cohort were recorded. Median OS, median PFS, hazard ratios (HRs) for OS and PFS by regional subgroups, and their 95% CIs were extracted.

### 2.4 Quality Assessment

The quality of enrolled studies was evaluated using the Cochrane Collaboration tool and scored through the following domains: selection bias, performance bias, detection bias, attrition bias, reporting bias and other biases (19). The risk level of each domain was rated as high, low or unclear. Publication bias was evaluated *via* funnel plots.

### 2.5 Statistical Analysis

First, extracted HRs and CIs from individual studies were pooled utilizing generic inverse variance. In studies that did not have a single “Asia” or “Western” subgroup, we used fixed effect models to generate the pooled estimates of region-specific survival HRs. Then, random effects models were used considering heterogeneity due to different trial designs, and forest plots were generated. Statistically significant heterogeneity was considered when *I* ^2^ > 50%. We also conducted an interaction test to determine the correlation of effect modifiers with regions and pooled HRs. Prespecified categories included line of therapy (first-line versus second-line or beyond) and combination strategy (monotherapy versus combination therapy).

Second, the correlation of median OS and median PFS from both experimental and control arms between Asian and Western populations was analyzed using a linear regression model, weighted by the sample size of each comparison. Pearson correlation coefficients (r) and their 95% CIs were calculated. In studies with more than one experimental arm, multiple separate comparisons were conducted.

All meta-analyses were conducted by RevMan 5.3 (The Cochrane Collaboration, Copenhagen, Denmark). Stata 16.0 (StataCorp, Texas, USA) was used for the regression analyses and subsequent graph plotting. All reported *P* values were 2-sided, and statistical significance was defined as *P* < 0.05.

## 3 Results

### 3.1 Study Selection and Characteristics

**Figure 1:**
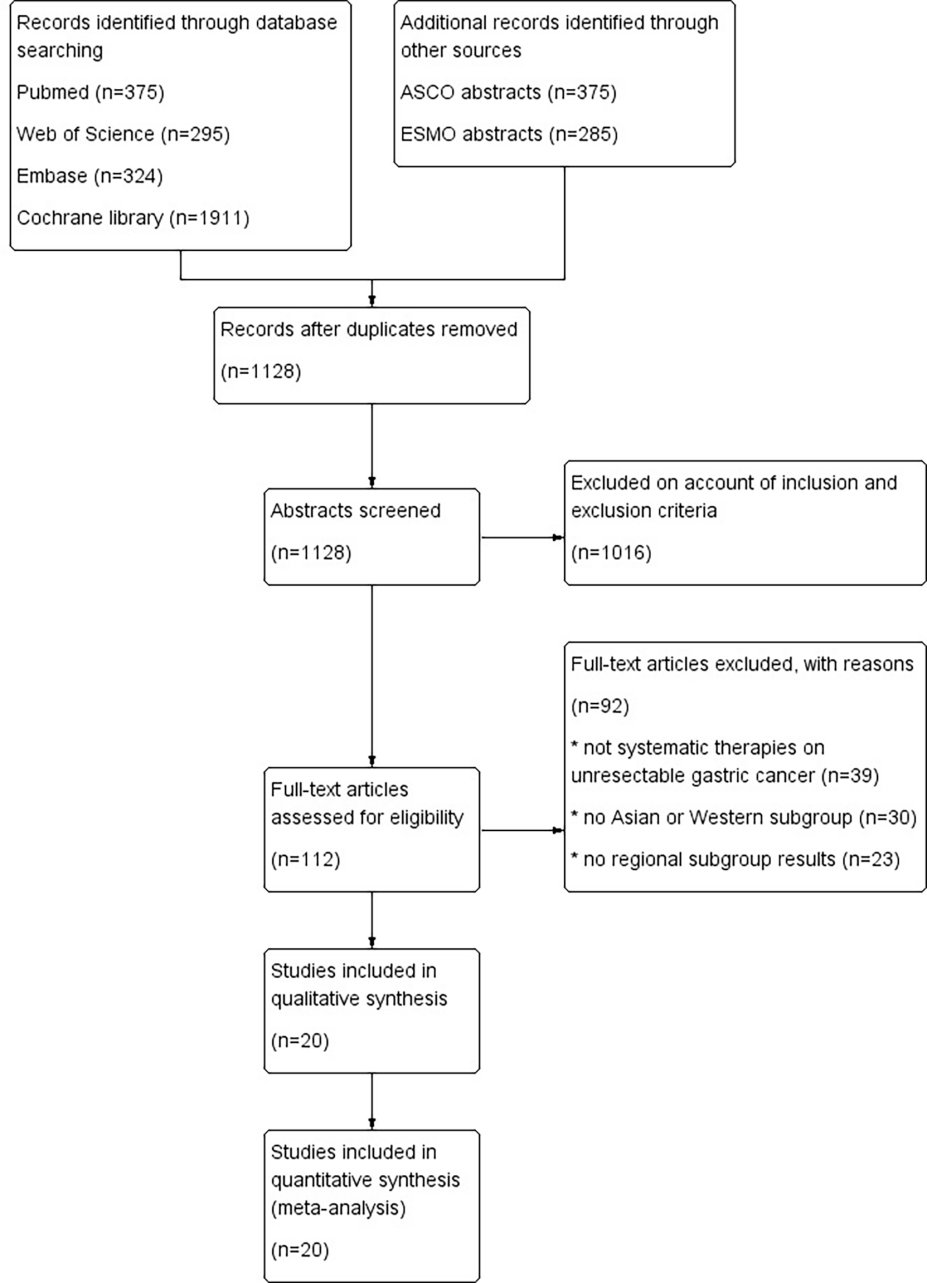
PRISMA flow diagram for the meta-analysis.

A total of 1654 potentially relevant publications were obtained from the literature search. After initial abstract review and duplicate removal, 20 original studies were considered eligible, comprising 9,033 patients for final analysis (**Figure 1**). The baseline characteristics of the 20 included studies are indicated in **Table 1**.

**Table 1:** Major characteristics of the eligible studies.

No.	Study Name	Year of publication	Name of first author	Treatment line	Phase	No. of Asian patients	No. of Western patients	Experimental arm	Control arm	Constituents of Asian populations	Constituents of Western populations
1	ToGA	2010	Yung-Jue Bang	1	III	319	190	CAPE/5-FU + DDP + Trastuzumab	CAPE/5-FU + DDP	China, Japan, South Korea	Europe
2	AVAGAST	2011	Atsushi Ohtsu	1	III	376	398	5-FU + DDP + BEV	5-FU + DDP	Japan, South Korea	Europe, Pan-America
3	EXPAND	2013	Florian Lordick	1	III	339	490	CAPE + DDP + Cetuximab	CAPE + DDP	South Korea	Europe
4	GRANITE-1	2013	Atsushi Ohtsu	2 or 2+	III	377	241	Everolimus	Placebo	China, Japan, South Korea	West Europe
5	REGARD	2014	Charles S Fuchs	2	III	26	245	RAM	Placebo	South Korea	North America, Europe, Australia, New Zealand
6	RAINBOW	2014	Hansjochen Wilke	2	III	223	398	PTX + RAM	PTX	South Korea, Japan, Taiwan, Hong Kong, Singapore	North America, West Europe
7	INTEGRATE	2016	Nick Pavlakis	1 or 2	II	54	93	Regorafenib	Placebo	South Korea	Canada, Australia, New Zealand
78	TRIO-013	2016	J. Randolph Hecht	1	III	193	17	CAPE + OXA + Lapatinib+	CAPE + OXA	China, South Korea, Hong Kong, Taiwan, Thailand	North America
9	GATSBY	2017	Peter C Thuss-Patience	2	III	157	188	Trastuzumab	PTX	Japan, South Korea	North America, West Europe
10	METGastric	2017	Manish A. Shah	1	III	183	379	5-FU + LV + OXA + Onartuzumab	5-FU + LV + OXA	South Korea, China	North America, Europe
11	N/A	2017	Yung-Jue Bang	1 MN	II	61	51	Ipilimumab	Placebo	South Korea	Europe
12	JACOB	2018	Josep Tabernero	1	III	369	266	Trastuzumab + DDP + CAPE/5-FU + Pertuzumab	Trastuzumab + DDP + CAPE/5-FU	Japan, China, South Korea, Taiwan, Thailand, Malesia	North America, West Europe
13	KEYNOTE-061	2018	Kohei Shitara	2	III	104	263	PEM	PTX	Japan, Hong Kong, South Korea, Taiwan, Malesia	North America, Europe, Australia, Israel
14	TAGS	2018	Kohei Shitara	3 or 3+	III	73	434	TAS-102	Placebo	Japan	North America, Europe
15	JAVELIN Gastric-300	2018	Yung-Jue Bang	3	III	93	278	Avelumab	PTX/IRI/Placebo	Japan, South Korea	Europe, North America, South America
16	RAINFALL	2019	Charles S Fuchs	1	III	69	520	5-FU + DDP + RAM	5-FU + DDP	Japan	Europe, Pan-America
17	N/A	2019	Yung-Jue Bang	1	III	86	67	LV + 5-FU + OXA + Ipatasertib	LV + 5-FU + OXA	South Korea, Singapore	North America, UK
18	KEYNOTE-062	2020	Kohei Shitara	1	III	123	295	DDP + 5-FU/CAPE + PEM	DDP + 5-FU/CAPE	Japan, South Korea	North America, Europe, Australia
19	JAVELIN Gastric-100	2020	Markus Moehler	1 MN	III	114	385	Avelumab	OXA + LV + 5-FU/CAPE	South Korea, Japan	North America, Europe (majority)
20	CheckMate-649	2020	Markus Moehler	1	III	236	135	OXA + LV + 5-FU/CAPE + NIVO	OXA + LV + 5-FU/CAPE	China, Japan, South Korea, Hong Kong, Taiwan, Singapore	North America

(MN, maintenance; CAPE, capecitabine; DDP, cisplatin; 5-FU, fluorouracil; BEV, bevacizumab; RAM, ramucirumab; PTX, paclitaxel; OXA, oxaliplatin; PEM, pembrolizumab; TAS-102, trifluridine/tipiracil; IRI, irinotecan; LV, leucovorin; NIVO, nivolumab).

Seventeen out of 20 studies were phase III clinical trials, and the remaining 3 were phase II trials. In terms of regional distribution, most trials were roughly balanced, while the TRIO-013 and REGARD trials predominantly enrolled Asian and Western participants, respectively. All 20 studies investigated nonconventional therapies: fourteen studies investigated the efficacy of targeted therapy (5 on VEGF, 4 on HER2, 1 on EGFR, 1 on MET, 1 on mTOR, and 1 on AKT); six studies focused on immunotherapy; and only one study explored the efficacy of a cytotoxic agent (TAS-102). Among all studies, eleven investigated first-line therapy, two investigated first-line maintenance therapy, and the remaining 7 were conducted at second- or later-line therapy. All trials were two-arm except KEYNOTE-062, which had a three-arm design.

### 3.2 Quality Assessments

All clinical trials conducted well-organized random sequence generation and allocation concealment (**Figure S1**). Eight trials did not blind the treatment allocation to participants or personnel, leading to a high risk of performance bias. Blinding of outcome assessment was not implemented in 7 trials, leading to risk of detection bias. No trial was at high risk of attrition and reporting bias. The funnel plots did not suggest significant publication bias (**Figure S2**).

### 3.3 Quantitative Analyses of the Overall Populations

Eighteen and 9 studies investigated the OS and PFS of systemic therapies stratified by region, respectively. Each of the included studies compared the efficacy of certain types of systemic therapy with standard-of-care treatment. The HRs of individual studies and the pooled results are summarized in **Supplementary Figure 3**. The overall estimated HR for OS among Asian patients was 0.89 with a 95% CI of 0.80–0.99 with nonsignificant heterogeneity (I^2^ = 36%, P = 0.06, **Supplementary Figure 3A**), demonstrating an 11% reduction in the hazard of death credited with experimental treatment. Similarly, among Western patients, our meta-analysis indicated that experimental treatment could decrease the risk of death by 14% (HR = 0.86; 95% CI, 0.80–0.93, **Supplementary Figure 3B**) without interstudy heterogeneity (I^2^ = 21%, P = 0.20).

The HRs for PFS of the individual studies and the pooled results are summarized in **Supplementary Figure 4**. In contrast with OS, our meta-analysis failed to suggest any survival benefit of experimental treatment versus control in terms of PFS in both Asian and Western patients (HR = 0.71; 95% CI, 0.48–1.04; HR = 0.80; 95% CI, 0.62–1.04 for Asian and Western patients, respectively).

### 3.4 Comparison of Efficacy Between Asian and Western Patients Stratified by Treatment Type

**Figure 2:**
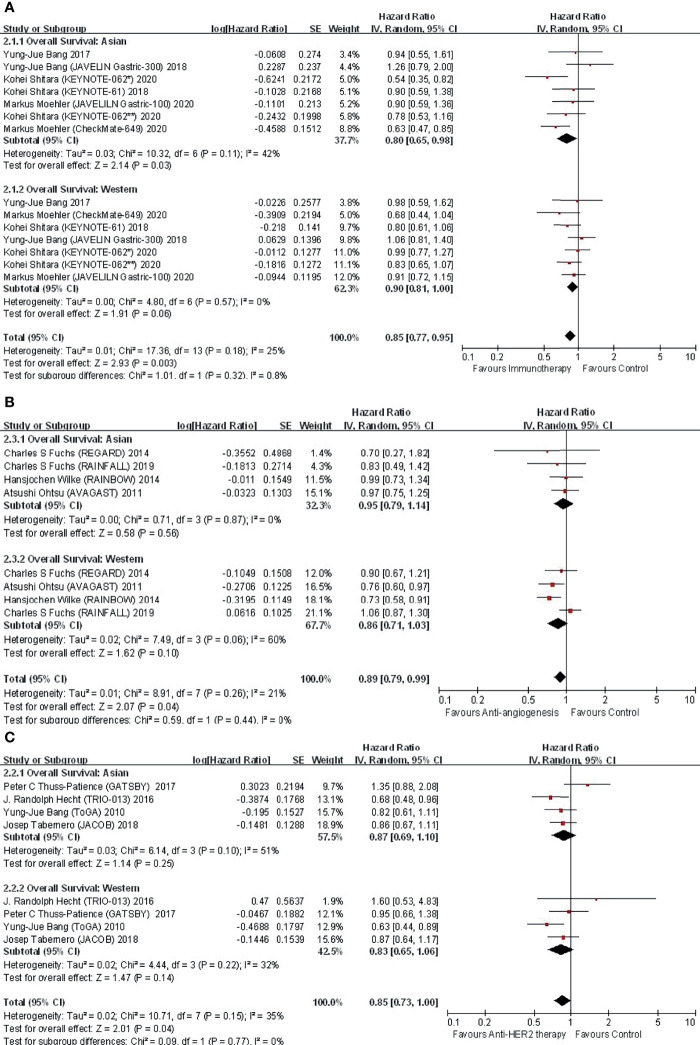
Comparison of regional subgroup differences in OS benefit with **(A)** immunotherapy; **(B)** anti-angiogenic therapy; and **(C)** anti-HER2 therapy.

In view that most of the included studies explored targeted therapy and immunotherapy where HRs for OS stratified by region were accessible, we pooled these data to make interregional comparisons classified by treatment categories (**Figure 2**). It should be mentioned that published subgroup data regarding HRs for PFS were incomplete. PFS was generally a secondary endpoint in clinical trials; therefore, the relevant subgroup analyses were frequently unimplemented. In that case, we did not perform the same analyses on PFS.

#### 3.4.1 Immunotherapy: Overall

In the 6 studies focusing on immunotherapy that reported subregional OS, there was no difference in OS between Asian and Western (P for interaction = 0.32) patients (**Figure 2A**). The application of immunotherapy elicited an improvement in OS in both Asians (HR, 0.80; 95% CI, 0.65–0.98) and Westerns (HR, 0.90; 95% CI, 0.81–1.00) compared with controls.

#### 3.4.2 Immunotherapy: Line of Therapy

**Figure 3:**
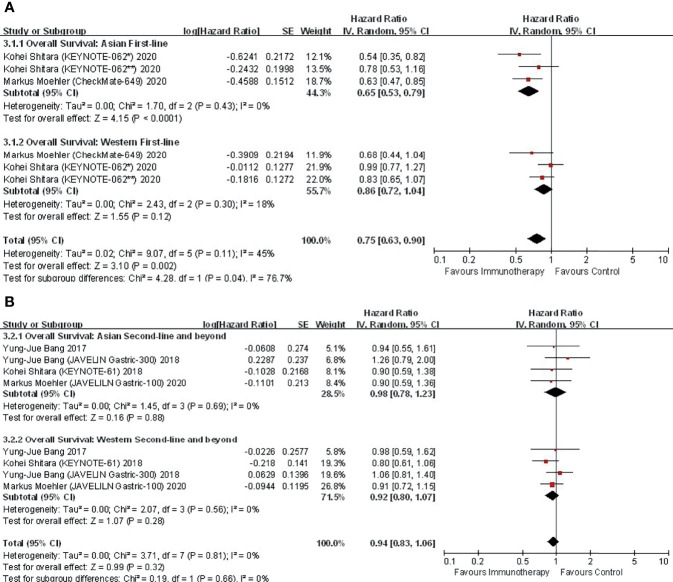
Comparison of regional subgroup differences in OS according to first versus subsequent lines of immunotherapy. **(A)** first-line; **(B)** second-line and beyond.

Among trials investigating immunotherapy, two were conducted in the first-line setting. Further subgroup analysis by treatment line in terms of OS suggested significant interregional differences (**Figure 3**). In the first-line setting, Asians displayed an improved OS (HR, 0.65; 95% CI, 0.53–0.79), while Westerns did not gain such benefit (HR, 0.86; 95% CI, 0.72–1.04), with P for interaction = 0.04 (**Figure 3A**). In regard to second-line treatment or beyond, neither Asian (HR, 0.98; 95% CI, 0.78–1.23) nor Western (HR, 0.92; 95% CI, 0.83–1.06) patients derived survival benefits from immunotherapy (P for interaction = 0.66) (**Figure 3B**).

#### 3.4.3 Immunotherapy: Combination Strategy

Among trials investigating immunotherapy, five investigated monotherapies, while two sought combination therapy (immunotherapy or immunotherapy plus chemotherapy were compared with chemotherapy alone in KEYNOTE-062) (**Supplementary Figure 5**). In the monotherapy subgroup, neither Asian (HR, 0.87; 95% CI, 0.66–1.14) nor Western (HR, 0.94; 95% CI, 0.83–1.06) patients exhibited a prolonged OS, indicating limited interregional variance (P for interaction = 0.60). Instead, in the combination subgroup, combination immunotherapy significantly improved OS in both Asian (HR, 0.56; 95% CI, 0.54–0.87) and Western (HR, 0.77; 95% CI, 0.63–0.93) patients (P for interaction = 0.22).

#### 3.4.4 Anti-Angiogenic Therapy: Overall

In the 4 studies focusing on antiangiogenic therapy that reported subgroup data, the OS benefit in Asian and Western patients was proportional (P for interaction = 0.44) (**Figure 2B**). Compared with the control, antiangiogenic therapy was not superior in either Asian (HR, 0.95; 95% CI, 0.79–1.14) or Western (HR, 0.86; 95% CI, 0.71–1.03) patients. Nevertheless, a trend of OS benefit with antiangiogenic therapy was shown.

#### 3.4.5 Anti-Angiogenic Therapy: Line of Therapy

Among trials investigating antiangiogenic therapies, two were conducted in the first-line setting, while the remaining two were conducted in the second-line setting (**Supplementary Figure 6**). Neither Asian (HR, 0.94; 95% CI, 0.75–1.19) nor Western (HR, 0.91; 95% CI, 0.66–1.26) patients yielded a survival benefit from first-line treatment (P for interaction = 0.85). In terms of second-line treatment, Western patients receiving antiangiogenic agents indicated prolonged survival (HR, 0.79; 95% CI, 0.64–0.97), while Asians (HR, 0.96; 95% CI, 0.72–1.28) did not, although the interregional difference was not statistically significant (P for interaction = 0.29).

#### 3.4.6 Anti-HER2 Therapy: Overall

In the 4 studies focusing on anti-HER2 therapy that reported subregional OS, anti-HER2 therapy did not significantly improve OS in either Asian (HR, 0.87; 95% CI, 0.69–1.10) or Western (HR, 0.83; 95% CI, 0.65–1.06) populations, although a trend of survival benefit with anti-HER2 therapy versus control was yielded (**Figure 2C**). Interregional disparity was not discovered (P for interaction =0.77). Subgroup analyses were unable to be carried out due to similar designs.

### 3.5 Comparison and Correlation Between Survival Parameters in Asian and Western Populations

Eleven eligible studies provided 22 pairs of median OS data for Asian and Western patients, consisting of 11 experimental arms and 11 control arms. The bar chart comparing median OS between Asian and Western populations is presented in **Supplementary Figure 7A**. The correlation of the median OS between Asian and Western patients was strong and statistically significant (r = 0.867, p < 0.001; **Supplementary Figure 7B**). According to the weighted linear regression analysis and the scatter plot, the majority of dots were located beyond the reference line y=x, indicating that Asian patients had a longer OS than Western patients.

Five eligible studies provided 10 pairs of median PFS data for Asian and Western patients, consisting of 5 experimental arms and 5 control arms. The bar chart comparing the median PFS between Asian and Western populations is presented in **Supplementary Figure 8A**. The correlation of the median PFS between Asian and Western patients was strong and statistically significant (r = 0.942, p < 0.001; **Supplementary Figure 8B**). According to the weighted linear regression analysis and the scatter plot, the dots were uniformly distributed on both sides of the reference line y=x, suggesting that Asian patients had a similar PFS to Western patients.

## 4 Discussion

Asian race has long been considered a favorable prognostic factor of late-stage GCs (4, 11, 20). Previous clinical studies mainly considered chemotherapy (11, 21). Nevertheless, as novel treatment means for advanced/metastatic GC/GEA are applied in clinical practice, whether they act consistently between Asian and Western patients remains undefined because confounding factors often impede researchers from making direct comparisons of intertrial numerical data.

This is the first meta-analysis that comprehensively compares the efficacy of multiple therapies in patients with unresectable GC or GEA from different regions. Our meta-analysis of 20 clinical trials indicates that both Asian and Western patients benefit from immunotherapy, anti-HER2, and anti-angiogenic therapies with no interregional differences in efficacy. Nonetheless, Asian patients benefit more from first-line immunotherapy in terms of OS. Asian patients with late-stage GC/GEA also have a remarkably longer OS than their Western counterparts.

The strength of our meta-analysis is the strict inclusion criteria that require both PFS and OS of regional subgroups in global trials, rather than solitary survival data. By pooling regional subgroup data from individual studies, we conducted more reliable comparisons where patients from different districts could be allocated evenly in each trial. Avoidance of direct comparison of survival data from single-site studies also considerably eliminated interstudy heterogeneity.

There are possible explanations for the little interregional differences of therapeutic effects with immunotherapy, anti-HER2 therapy and anti-angiogenic therapy. It has been acknowledged that responses to either immunotherapy or targeted therapy are biomarker determinative. From one perspective, hallmarks such as PD-L1 and HER2 are expressed equivalently among Asian and Western GCs (22). From another perspective, although there might be undiscovered factors exerting an impact on prognosis, the complex regulation system of the tumor microenvironment attenuates their single function. Multiple predictors could counteract one another, leading to similar treatment responses (23, 24).

Interestingly, Asian patients seem to be more sensitive to first-line immunotherapy, as suggested by the pooled results of two large RCTs, CheckMate-649 and KEYNOTE-062, which is in line with data from clinical trials regarding a few other cancer types (25, 26). However, it is challenging to interpret these results.

Previous studies have proposed the regional disparities of GC/GEA in clinicopathological characteristics. Proximal tumors are more common in Western patients, while antral tumors are dominant in Asians (4, 27). For Lauren classification, the proportion of intestinal-type is higher in Asians (4). In regard to molecular subtyping, the distribution proportion of 4 GC subtypes proposed by TCGA (EBV-positive, genomically stable, microsatellite instable and chromosomal instable) is similar in the East and the West, with Korea being an outlier at prominently higher rates of GCs being MSI- or EBV-positive (28–30). However, the somatic mutation or gene amplification rates of several driver genes, including APC, ARIDIA, PIK3CA, PTEN and KRAS, vary greatly across races (31). Genetic polymorphisms and epigenome properties are also regional (32, 33). Reportedly, the presence of certain oncogenic mutations or promotor alternations is associated with resistance to immunotherapy (34–36). In addition, the diversity of dietary structures between Asian and Western regions might affect constituents of gut microbiota, exerting an impact on the efficacy of immunotherapy in gastrointestinal cancers (37, 38). All these factors could account for the interregional disparity in sensitivity to immunotherapy.

On all accounts, the prognosis of GC/GEA should be judged with caution. According to the results of CheckMate-649 and KEYNOTE-062, responses to immunotherapy could not be explained merely by conventional indicators such as PD-L1 level or TMB. These two biomarkers are far from faultless for screening out immunotherapy-sensitive populations. A vast number of patients in CheckMate-649 and KEYNOTE-062 with PD-L1-negative or TMB-low tumors generated anomalous durable responses. Given that antitumor immunity differs across untreated and heavily treated patients, we boldly speculate that an undiscovered Asian signature might reside in a treatment-naïve immune context and favors first-line immunotherapy. This possible ethnic-specific signature could be exploited to assist prognosis stratification together with traditional indicators such as PD-L1 and TMB. From our perspective, the development of new hallmarks for predicting responses to immunotherapy must take into account the unique immunogenomic features that Asian and Western patients do not share (39, 40). As immune checkpoint inhibitors play an increasingly critical role in the treatment of multiple advanced cancers, the correlation between geographic locality and treatment responses warrants further investigations (41).

According to our results, Asian patients with unresectable GC/GEA present a longer OS than Western patients regardless of treatment type, which is highly in accordance with previous records. Intriguingly, PFS is similar across two populations. One possible explanation is that immunotherapy could impose a lasting antitumor effect in treatment-sensitive patients even after radiographic progression, leading to a longer post-progression survival. In addition, Asian patients with advanced/metastatic GC/GEA generally receive more cytotoxic therapies and palliative care after disease progression (evidenced in RAINBOW and AVAGAST trials), possibly contributing to a superior OS among Asians (12, 42). In addition, Asian patients show better baseline physical status than their Western counterparts, which might portend better tolerance and reactivity to subsequent therapies. However, the hypotheses presented above need to be examined in prospective studies with further analyses.

The limitations of our studies are as follows. First, due to the variation of disease prevalence, the definition of “Asian” or “Western” is inconsistent across studies. Although we regrouped the data of each trial, the scope of these two terms was not uniform in the strict sense. Second, eligible studies investigating cytotoxic agents are scarce. On the one hand, the standard treatment regimen of GC/GEA is different between the East and the West. Thus, it is difficult to conduct a chemotherapy trial with the same interventions on participants of different districts. On the other hand, a substantial number of excluded clinical trials did not perform subgroup analyses, making a portion of survival data inaccessible. Third, inclusion criteria with respect to biomarkers differ across studies, which is inevitable but could bias our pooled analyses (e.g., PD-L1 CPS ≥1 or CPS ≥5; HER2 IHC 3+ or 2+).

In conclusion, although Asian and Western patients with unresectable GC/GEA possess different clinical and genetic profiles, they respond similarly to systemic therapies with limited interregional differences. Exceptionally, Asian patients indicate a superior responsiveness to first-line immunotherapy. In addition, Asian patients also present a higher OS, rather than PFS, than Western patients. These results may be implicated in the design of multinational clinical trials. For example, if geographic heterogeneity of drug efficacy is found, research directors are amenable to determine the minimal sample size for each participant district, ensuring consistency of regional outcome in accordance with the global tendencies. This process could tremendously improve work efficiency and conserve resources, including time and funds.

## Data Availability Statement

The original contributions presented in the study are included in the article/**Supplementary Material**. Further inquiries can be directed to the corresponding author.

## Author Contributions

ZZ, ZL, and ZC contributed to conception and design of the study. ZZ and ZL conducted the literature search, extracted the data and performed the statistical analysis. ZZ wrote the first draft of the manuscript. ZL and ZC contributed to manuscript revision, read, and approved the submitted version.

## Conflict of Interest

The authors declare that the research was conducted in the absence of any commercial or financial relationships that could be construed as a potential conflict of interest.

## Publisher’s Note

All claims expressed in this article are solely those of the authors and do not necessarily represent those of their affiliated organizations, or those of the publisher, the editors and the reviewers. Any product that may be evaluated in this article, or claim that may be made by its manufacturer, is not guaranteed or endorsed by the publisher.

## Supplementary Material

Supplementary Figure 1 Risk of bias summary **(A)** and bias graph **(B).**

Supplementary Figure 2 Funnel plots: OS **(A)** and PFS **(B)** in Asian populations; OS **(C)** and PFS **(D)** in Western populations.

Supplementary Figure 3 HRs of OS in patients receiving systemic therapies versus controls in Asian and Western populations. Each study was shown by the study name and year of publication. For each trial, the position of the square denoted the HR value, horizontal lines represented 95% CIs, and diamond plots represented overall results. **(A)** HRs of OS in the Asian population; **(B)** HRs of OS in Western patients.

Supplementary Figure 4 Hazard ratios of PFS in patients receiving exploratory therapies versus controls in Asian and Western populations. Each study was shown by the study name and year of publication. For each trial, the position of the square denoted the HR value, horizontal lines represented 95% CIs, and diamond plots represented overall results. **(A)** HRs of PFS in the Asian population; **(B)** HRs of PFS in Western patients.

Supplementary Figure 5 Comparison of regional subgroup differences in OS according to single versus combination immunotherapy. **(A)** monotherapy; **(B)** combination therapy.

Supplementary Figure 6 Comparison of regional subgroup differences in OS according to first versus subsequent lines of antiangiogenic therapy. **(A)** first-line; **(B)** second-line.

Supplementary Figure 7 Bar chart **(A)** and scatter plot with linear regression analysis **(B)** of median OS between Asian and Western patients. Pearson correlation coefficients (r) and correlation equations are shown. The red line represents the reference line y=x, suggesting equivalent OS between Asian and Western patients.

Supplementary Figure 8 Bar chart **(A)** and scatter plot with linear regression analysis **(B)** of median PFS between Asian and Western patients. Pearson correlation coefficients (r) and correlation equations are shown. The red line represents the reference line y=x, suggesting equivalent PFS between Asian and Western patients.
