# Supplementary figures and images for: Comparison of Treatment Efficacy and Survival Outcomes Between Asian and Western Patients With Unresectable Gastric or Gastro-Esophageal Adenocarcinoma: A Systematic Review and Meta-Analysis
Source: Front Oncol. 2022 Mar 7;12:831207. doi: 10.3389/fonc.2022.831207 (PMC8936077; doi:10.3389/fonc.2022.831207)

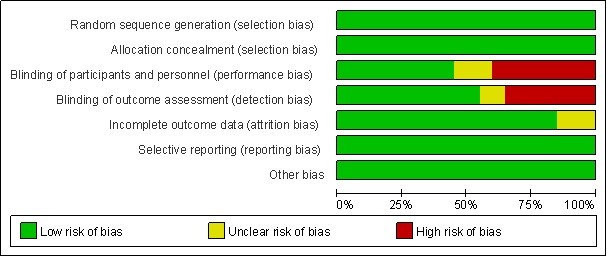

Supplement: Supplementary Figure 1 — Risk of bias summary (A) and bias graph (B). [file Image_1.jpeg]

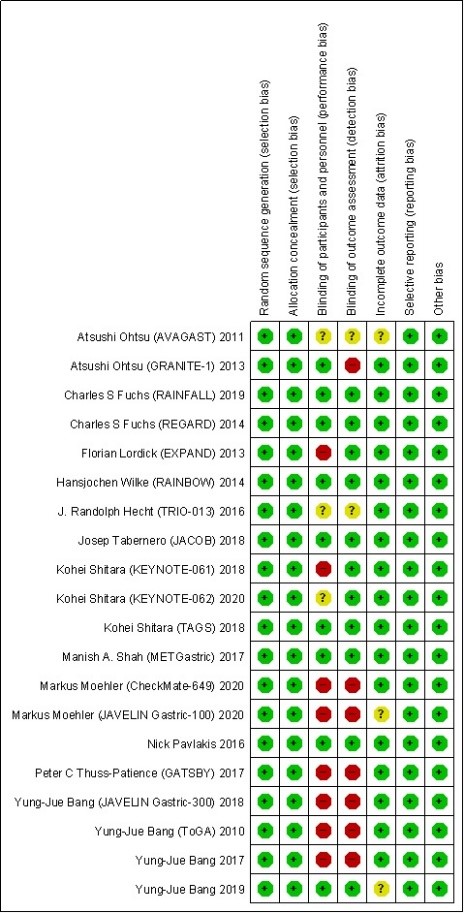

Supplement: Supplementary Figure 2 — Funnel plots: OS (A) and PFS (B) in Asian populations; OS (C) and PFS (D) in Western populations. [file Image_2.jpeg]

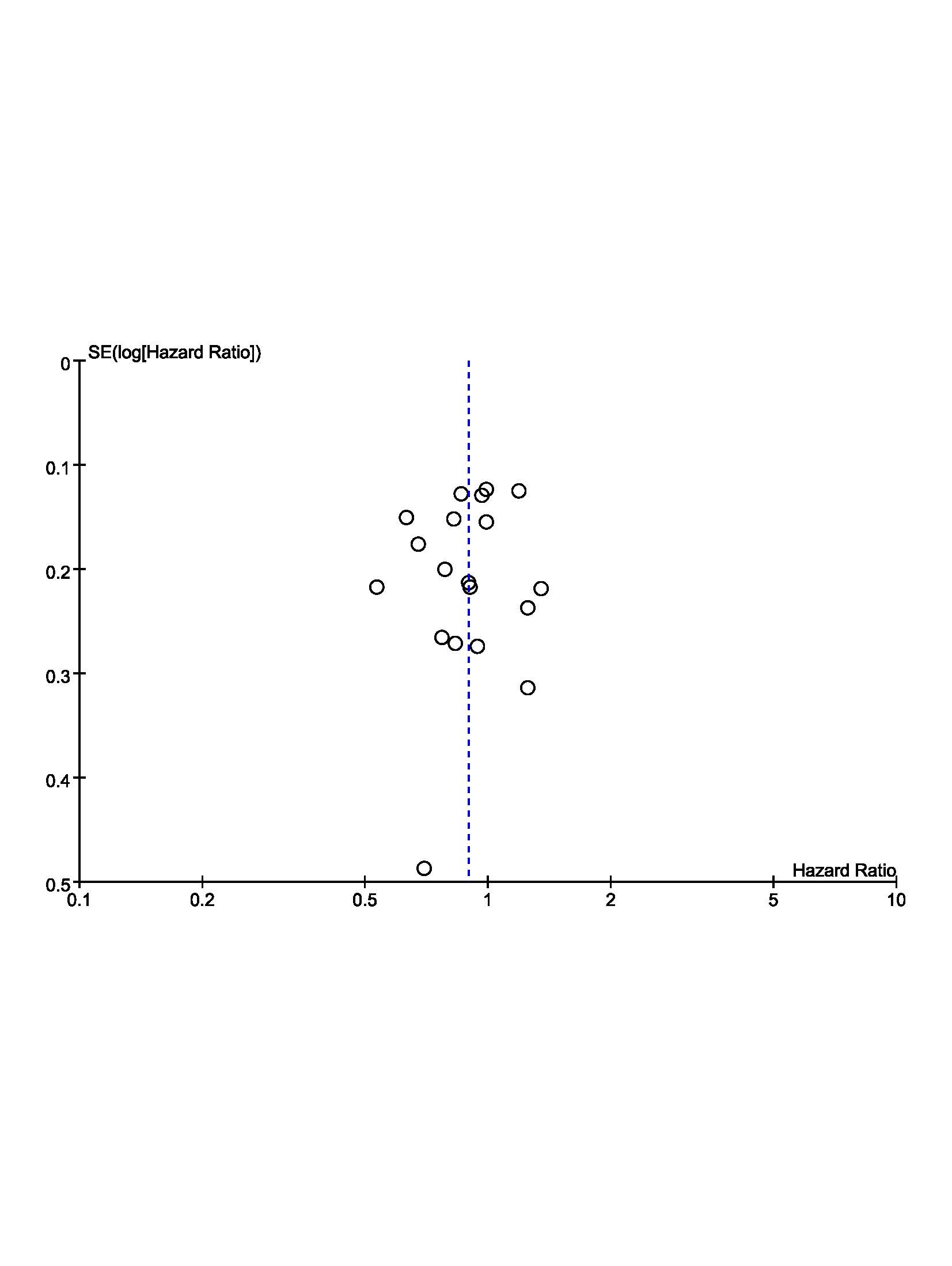

Supplement: Supplementary Figure 3 — HRs of OS in patients receiving systemic therapies versus controls in Asian and Western populations. Each study was shown by the study name and year of publication. For each trial, the position of the square denoted the HR value, horizontal lines represented 95% CIs, and diamond plots represented overall results. (A) HRs of OS in the Asian population; (B) HRs of OS in Western patients. [file Image_3.jpeg]

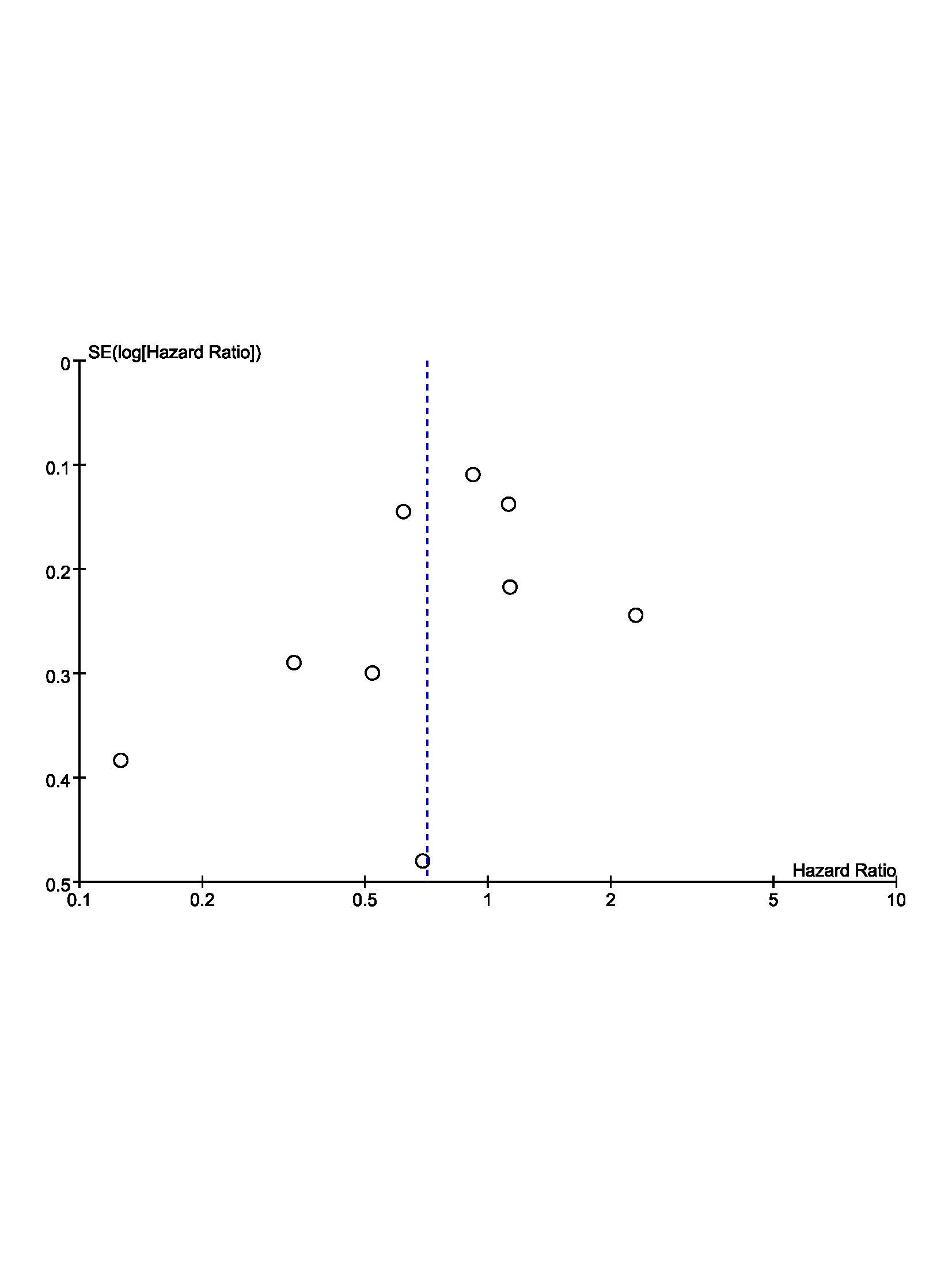

Supplement: Supplementary Figure 4 — Hazard ratios of PFS in patients receiving exploratory therapies versus controls in Asian and Western populations. Each study was shown by the study name and year of publication. For each trial, the position of the square denoted the HR value, horizontal lines represented 95% CIs, and diamond plots represented overall results. (A) HRs of PFS in the Asian population; (B) HRs of PFS in Western patients. [file Image_4.jpeg]

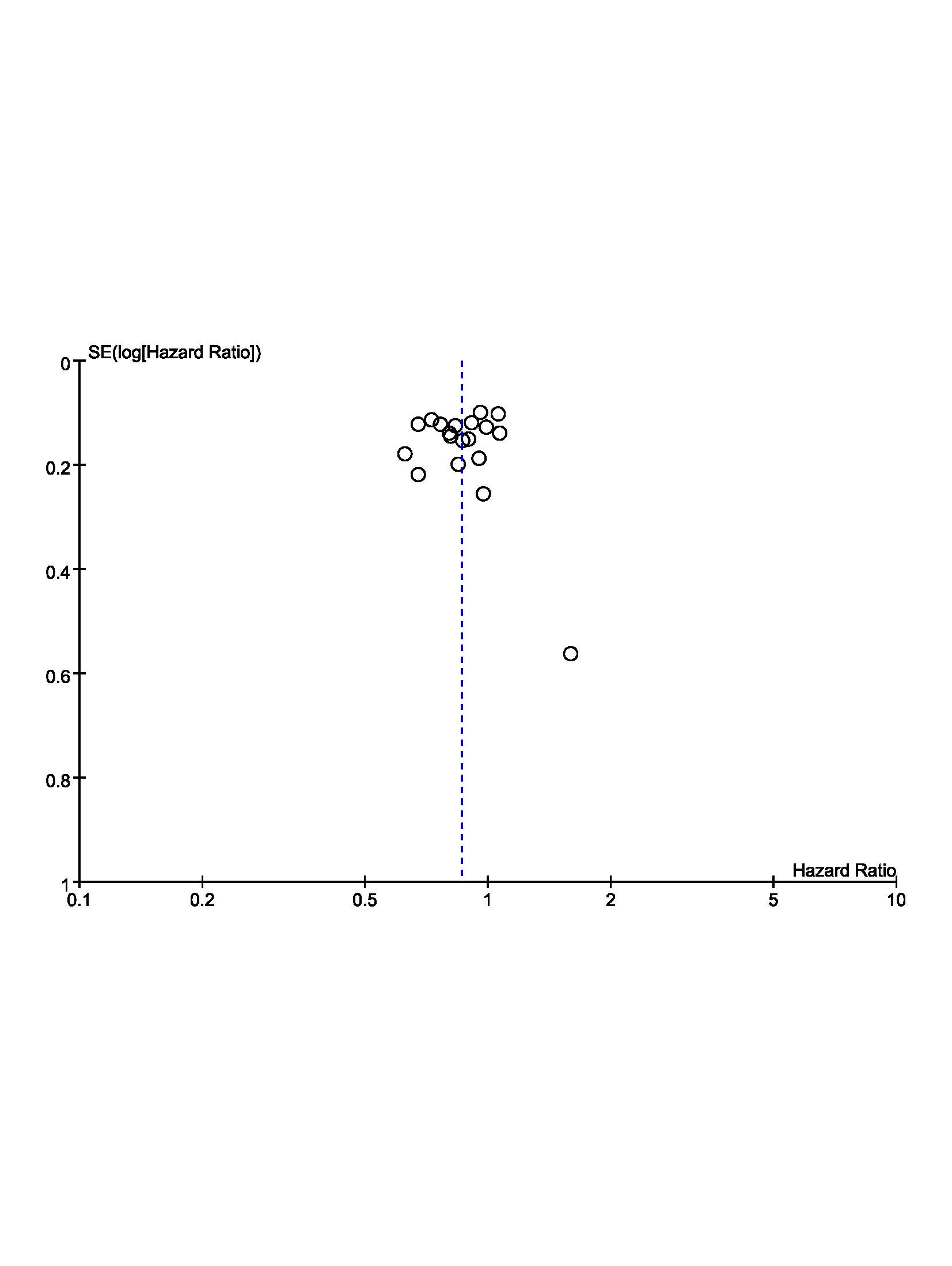

Supplement: Supplementary Figure 5 — Comparison of regional subgroup differences in OS according to single versus combination immunotherapy. (A) monotherapy; (B) combination therapy. [file Image_5.jpeg]

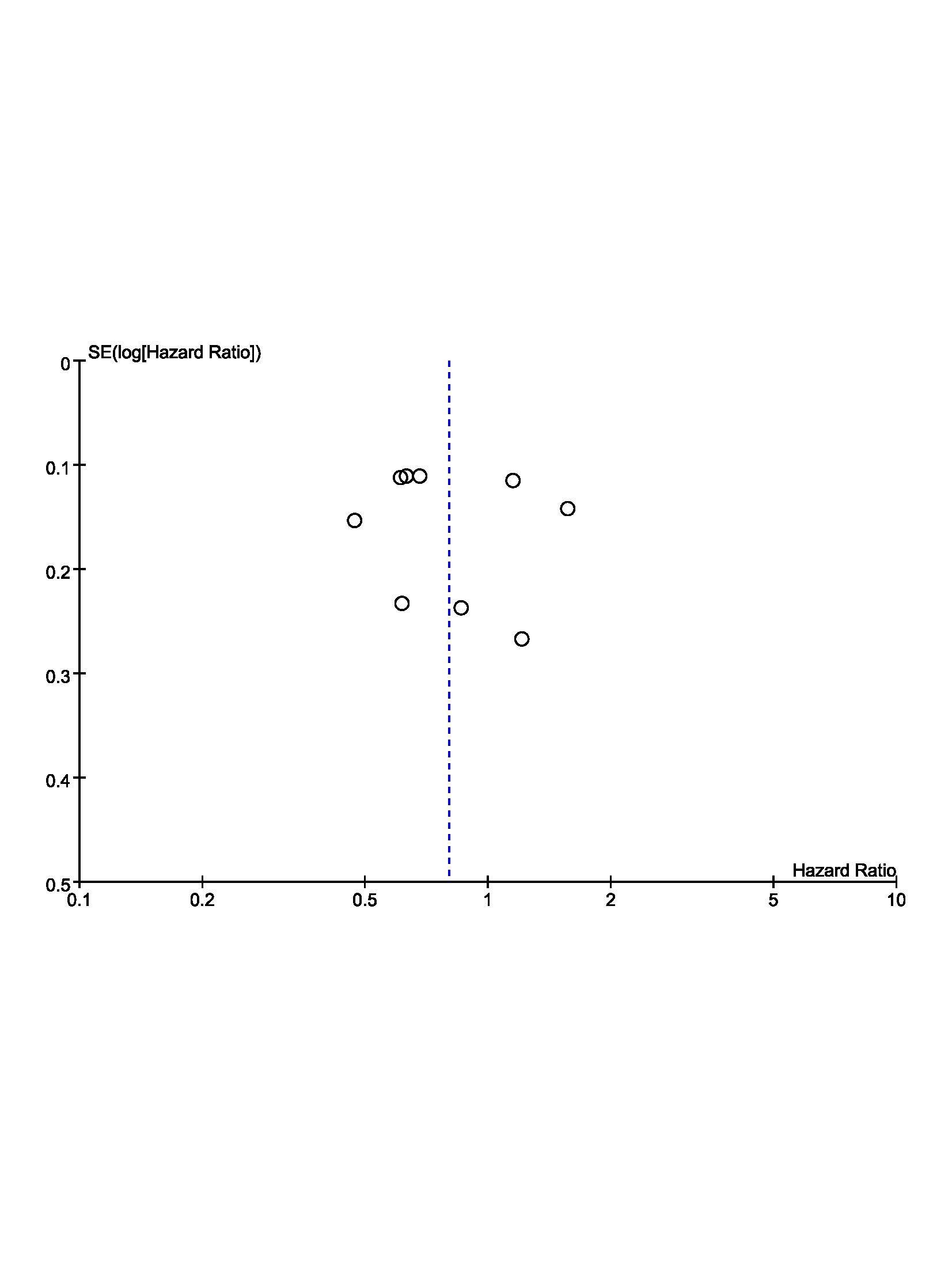

Supplement: Supplementary Figure 6 — Comparison of regional subgroup differences in OS according to first versus subsequent lines of antiangiogenic therapy. (A) first-line; (B) second-line. [file Image_6.jpeg]

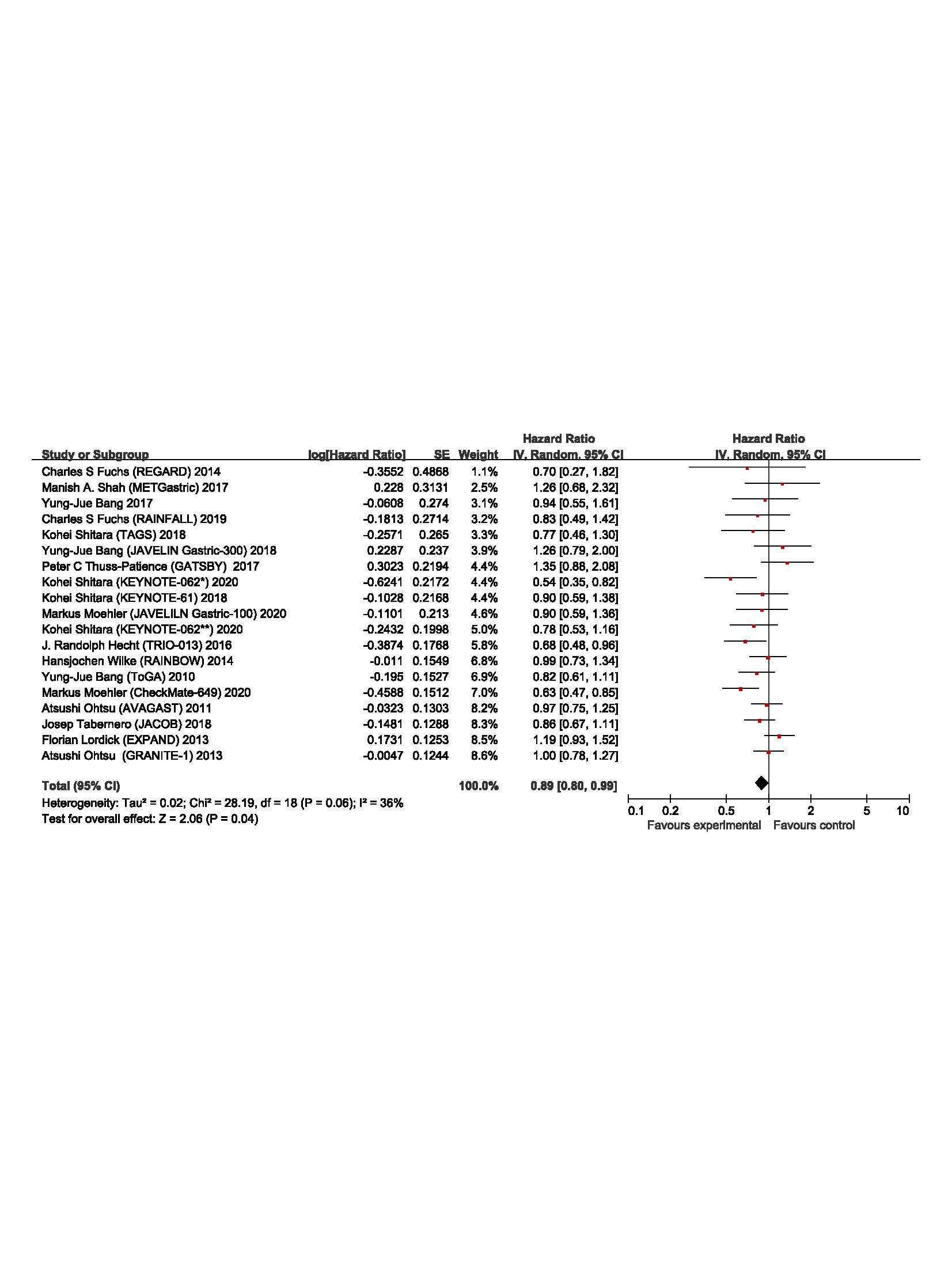

Supplement: Supplementary Figure 7 — Bar chart (A) and scatter plot with linear regression analysis (B) of median OS between Asian and Western patients. Pearson correlation coefficients (r) and correlation equations are shown. The red line represents the reference line y=x, suggesting equivalent OS between Asian and Western patients. [file Image_7.jpeg]

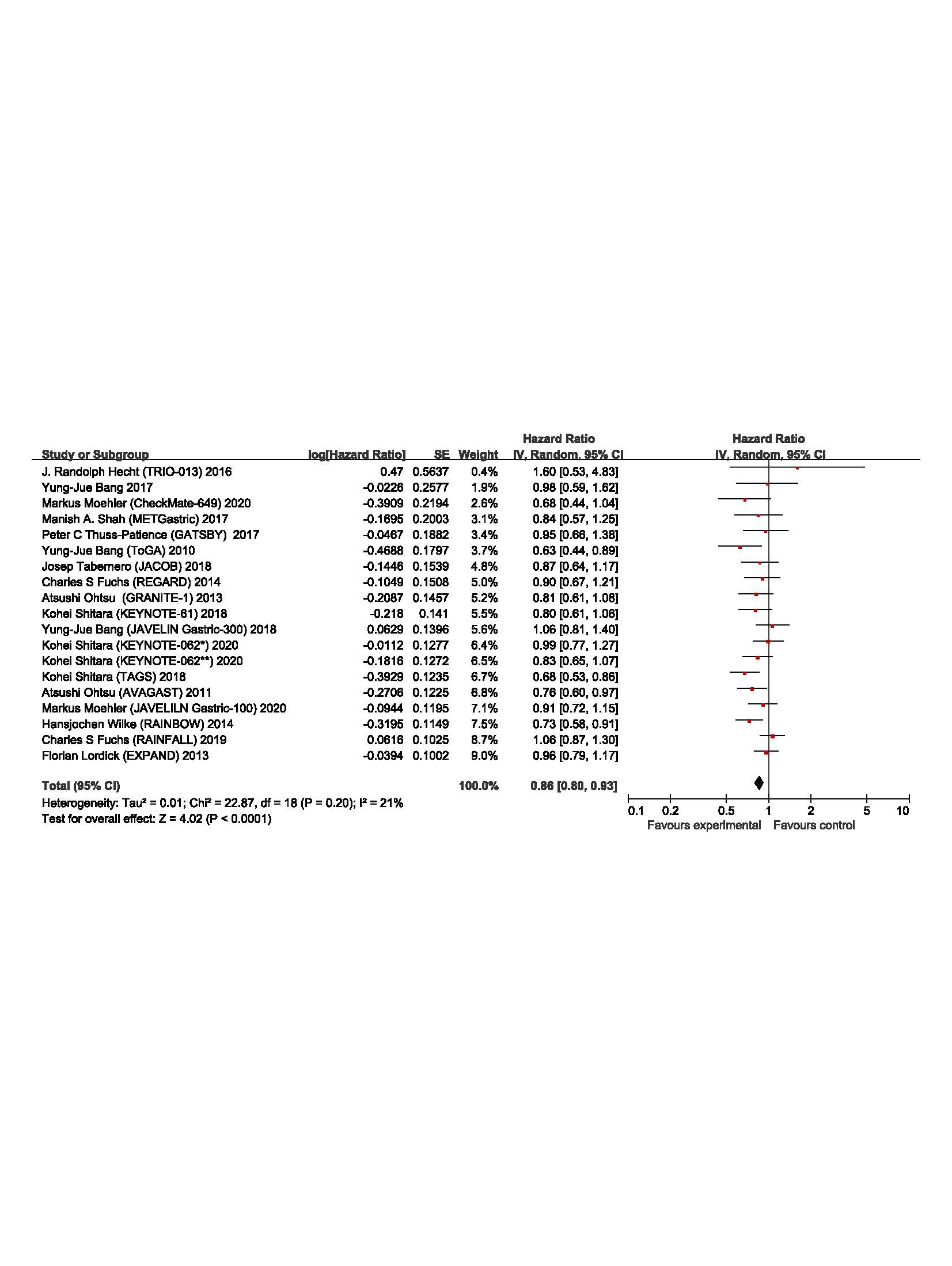

Supplement: Supplementary Figure 8 — Bar chart (A) and scatter plot with linear regression analysis (B) of median PFS between Asian and Western patients. Pearson correlation coefficients (r) and correlation equations are shown. The red line represents the reference line y=x, suggesting equivalent PFS between Asian and Western patients. [file Image_8.jpeg]

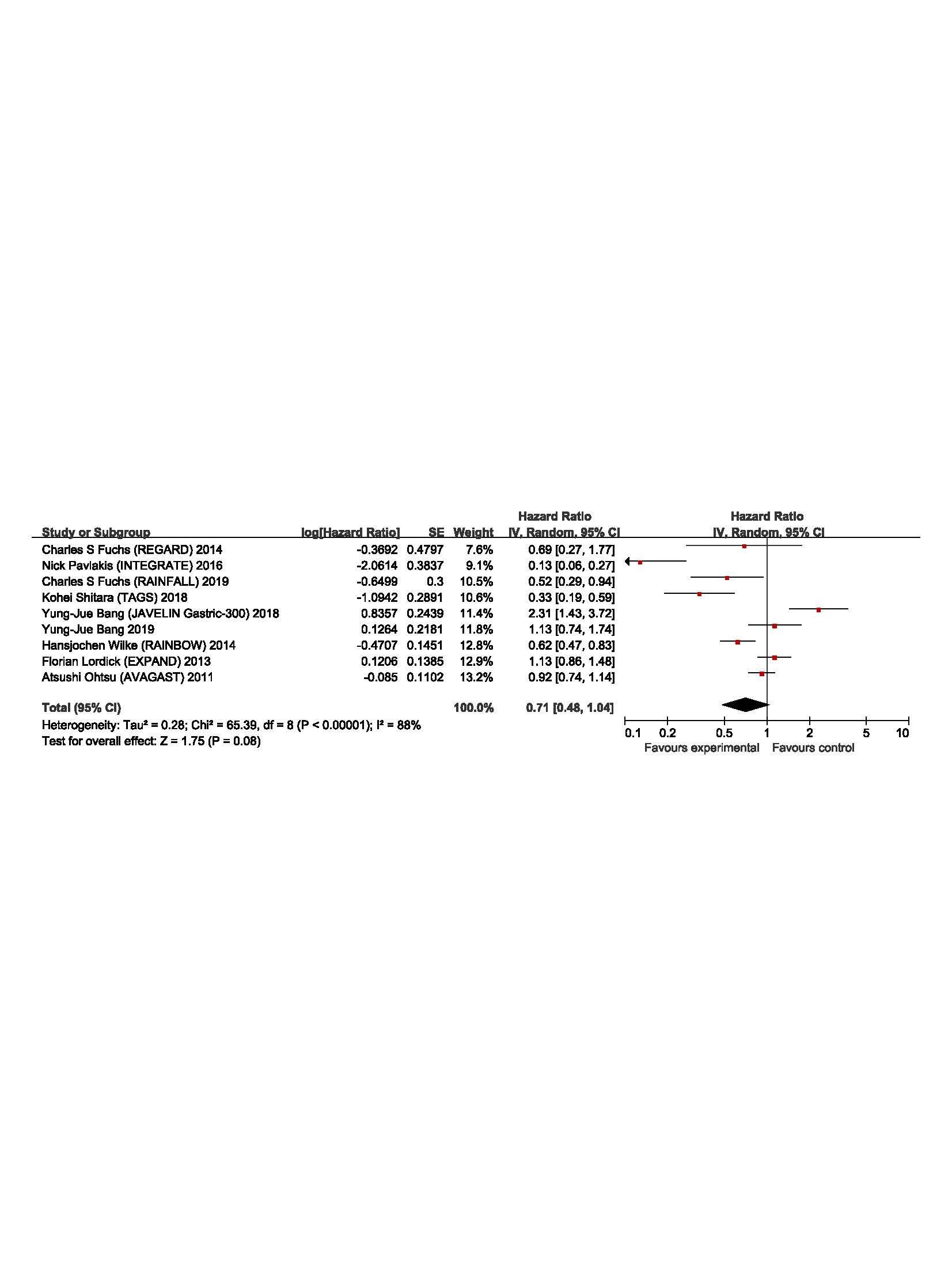

Supplement: Supplementary file 9 [file Image_9.jpeg]

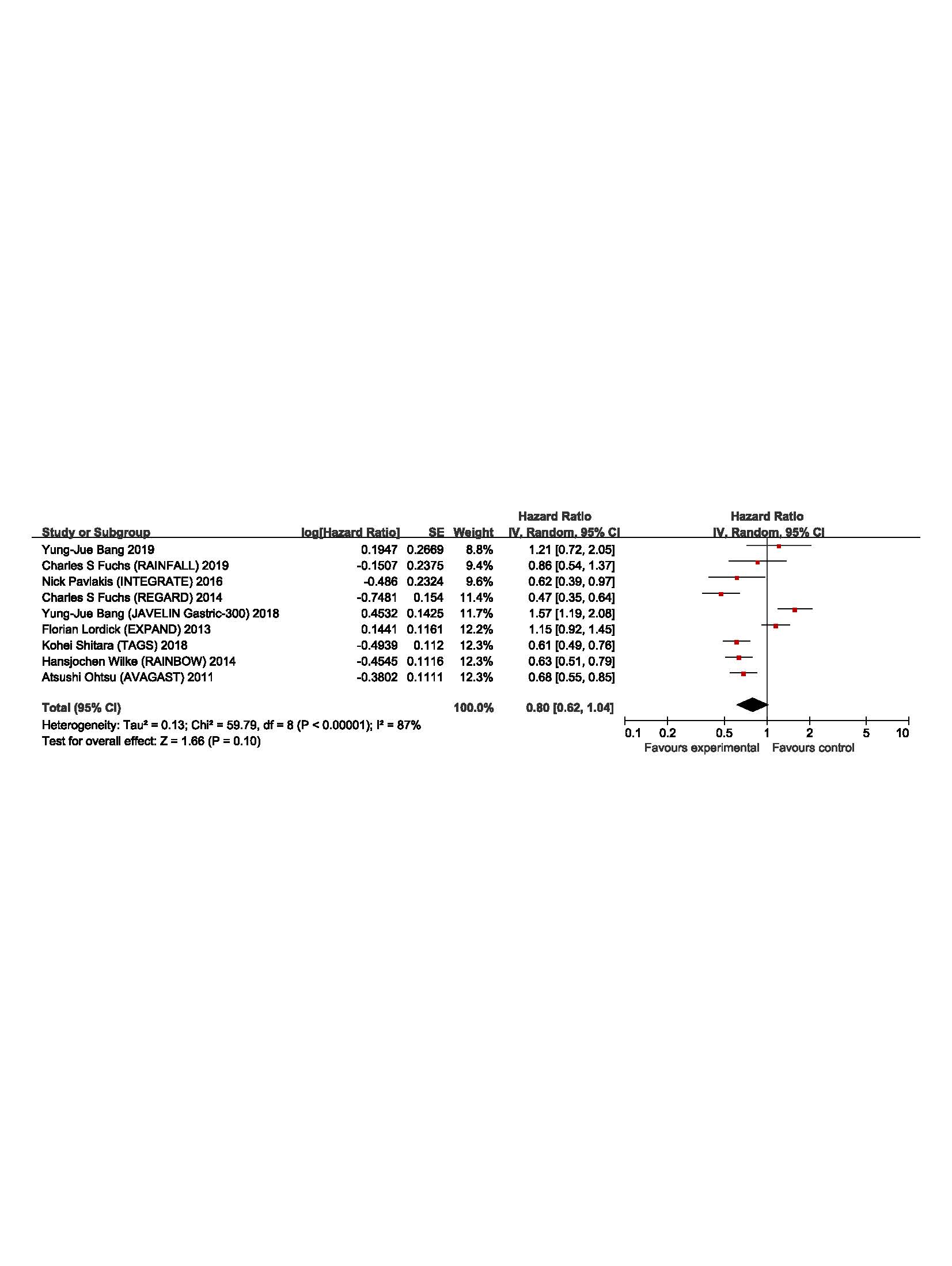

Supplement: Supplementary file 10 [file Image_10.jpeg]

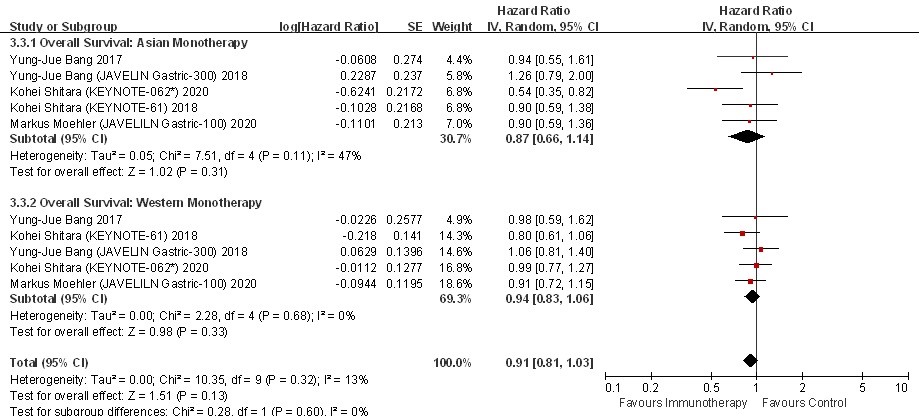

Supplement: Supplementary file 11 [file Image_11.jpeg]

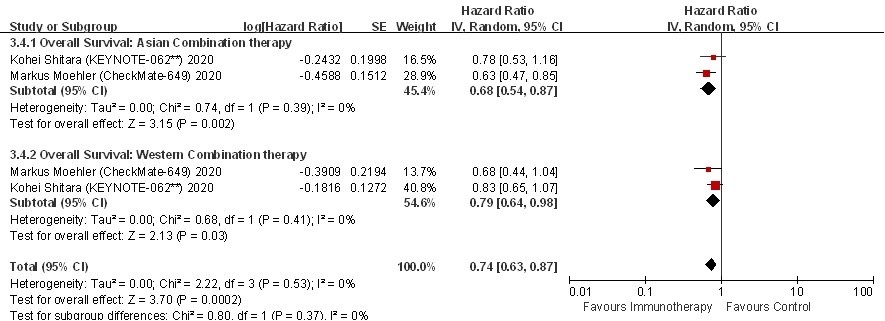

Supplement: Supplementary file 12 [file Image_12.jpeg]

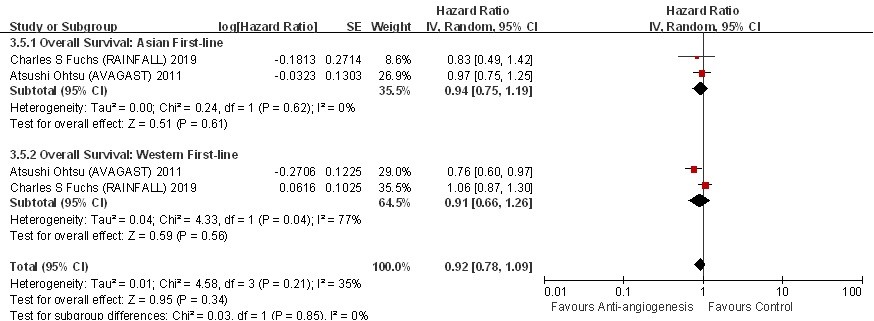

Supplement: Supplementary file 13 [file Image_13.jpeg]

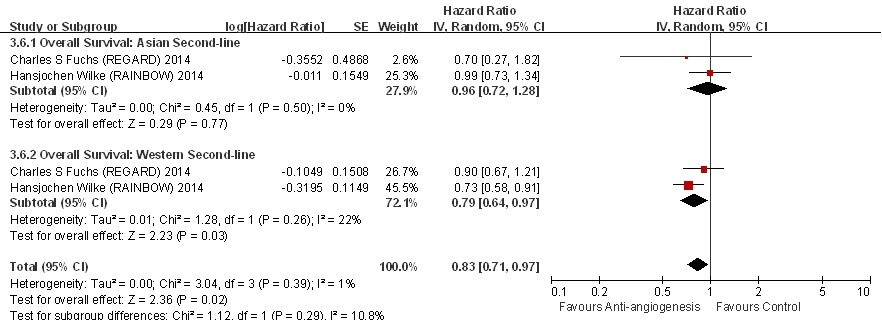

Supplement: Supplementary file 14 [file Image_14.jpeg]
