# Supplementary material for: Comparison of Treatment Efficacy and Survival Outcomes Between Asian and Western Patients With Unresectable Gastric or Gastro-Esophageal Adenocarcinoma: A Systematic Review and Meta-Analysis
Source: Front Oncol. 2022 Mar 7;12:831207. doi: 10.3389/fonc.2022.831207 (PMC8936077; doi:10.3389/fonc.2022.831207)
Supplement: Supplementary file 15 [file Table_1.docx]

**Table S1 Search algorithm.**

| Database or engine | Search statement | Filter |
| --- | --- | --- |
| PubMed-MEDLINE | (((gastric cancer[MeSH Terms]) OR (gastroesophageal cancer[MeSH Terms]) OR (gastric adenocarcinoma[Title/Abstract])) AND ((Asia[Title/Abstract]) OR (Asian[Title/Abstract]) OR (multiregional[Title/Abstract]) OR (global[Title/Abstract]) OR (international[Title/Abstract]) OR (multinational[Title/Abstract]) OR (multicenter[Title/Abstract])) AND ((drug therapy[MeSH Subheading]))) OR (((gastric cancer[MeSH Terms]) OR (gastroesophageal cancer[MeSH Terms]) OR (gastric adenocarcinoma[Title/Abstract])) AND ((subgroup[Title/Abstract]) OR (subanalysis[Title/Abstract]) OR (subpopulation[Title/Abstract]) OR (subcohort[Title/Abstract])) AND ((Asia[Title/Abstract]) OR (Asian[Title/Abstract])) AND ((drug therapy[MeSH Subheading]))) OR (((gastric cancer[MeSH Terms]) OR (gastroesophageal cancer[MeSH Terms]) OR (gastric adenocarcinoma[Title/Abstract])) AND ((phase 3[Title/Abstract]) OR (phase III[Title/Abstract])) AND ((drug therapy[MeSH Subheading]))) | Clinical trial |
| Web of Science | TS=(advanced OR unresectable OR metastatic OR recurrent) AND TI=(gastric cancer  OR gastric adenocarcinoma OR gastroesophageal)  AND TS=(international  OR global  OR multicenter  OR multicenter  OR Asian OR multinational OR multiregional) | Clinical trial |
| Embase | gastric AND cancer:ab, ti AND (advanced:ab, ti OR unresectable:ab, ti OR metastatic:ab, ti) AND (multicohort:ab, ti OR multinational:ab, ti OR multiregional:ab, ti OR global:ab, ti OR international:ab, ti OR asian:ab, ti OR asia:ab, ti) AND [controlled clinical trial]/lim AND [english]/lim | Controlled trial |
| Cochrane library | (gastric cancer):ti, ab,kw AND (advanced):ti OR (metastatic):ti AND (Asian):ti, ab,kw AND (subgroup):ti, ab,kw | Trials |
| ASCO | Gastric cancer; gastroesophageal cancer | N/A |
| ESMO | Gastric cancer; gastroesophageal cancer | N/A |

(MeSH, Medical Subject Headings; N/A, not applicable)
